# Women’s experience of intimate partner violence and uptake of Antenatal Care in Sofala, Mozambique

**DOI:** 10.1371/journal.pone.0217407

**Published:** 2019-05-24

**Authors:** Halkeno Tura, Armando Licoze

**Affiliations:** 1 Department of Community and Behavioral Health, College of Public Health, University of Iowa, Iowa City, Iowa, United States of America; 2 Oasis Mozambique, Sofala, Mozambique; University of Cape Town, SOUTH AFRICA

## Abstract

Intimate partner violence (IPV) is associated with negative physical and mental health outcomes. Although maternal health services, especially antenatal care (ANC), can act as a link to IPV resources, women experiencing IPV likely have reduced uptake of ANC due to social and emotional barriers. Poor ANC uptake can also further exacerbate adverse pregnancy outcomes. However, there is limited research examining the association between IPV and ANC within the context of Mozambique. Using data from a study conducted to assess the impact of membership in savings groups on maternal health service utilization in Mozambique (N = 205), we investigated the association between IPV and uptake of ANC. Pearson chi-square and logistic regression were employed to examine the association between IPV and ANC service utilization. The mean age of the participants was 33.4 years (SD = 11.88). Overall, 47.3%, 83.4%, and 51.7% of the participants reported experiencing IPV, receiving at least one ANC, and four or more ANC, respectively. Women who reported experience of IPV had lower odds of receiving both at least one (AOR 0.31 [95% CI:0.12–0.82]) and four or more ANC (AOR 0.50 [95% CI: 0.27–0.92]). Women who reported experience of IPV also had lower odds of receiving ANC from skilled personnel (AOR 0.32 [95% CI: 0.10–0.90]). Experience of IPV showed significant association with reduced ANC service utilization among women in the study area. Further study is needed to assess whether the negative association between IPV and ANC service utilization is also a causal relationship, the evidence which will then help guide a comprehensive intervention effort to improve maternal health services use.

## Introduction

Intimate Partner Violence (IPV) against women is a major public health problem and the most frequent form of violence against women [1–3]. Globally, it is estimated that about 30% of women who have ever been in a relationship report that they have experienced some form of violence (e.g. a threat of physical, sexual violence, and/or psychological/emotional abuse) by their intimate partner [4]. Women in low and middle-income countries carry the most burden of IPV, and the prevalence of self-reported IPV in these settings ranges from 30% in Central and South America [5] to nearly 70% in sub-Saharan Africa [4].

In Mozambique, 40% of ever-partnered women of reproductive age (15–49) reported experience of physical, sexual or emotional violence perpetuated by an intimate male partner within 12 months preceding the interview [6]. In a nationally representative survey [6], 27% of women reported that their male partners used emotional abuse to maintain control over them. A study conducted by a forensic physician/pathologist showed that 45% of all injuries experienced by women in Mozambique’s capital Maputo, were related to domestic violence and of all cases of domestic violence 78% was perpetuated by male partner [7].

In Mozambique, IPV is not only indicative of a deep-rooted gender inequality, but also a phenomenon that is culturally accepted rather than challenged. For example, the most recent demographic and health survey showed that one in four women and one in five men reported that wife beating is justified for at least one specific reason [6]. Study showed that the cultural norm in most of the community groups is to not only tolerate violence against women, but also to expect women to be submissive to men where the violation of such norms results in abuse by male partners [7].

Health outcomes among IPV victims may follow three possible pathways [5]. First, poor health outcomes may be due to injuries such as musculoskeletal or soft tissue damage resulting from experience of IPV. Second, coercion and male partners’ controlling behaviors may negatively affect women’s health by reducing autonomy, especially when male partners use controlling behaviors to limit their partners’ ability to make decisions about their healthcare. A third pathway posits that experiencing IPV can lead to negative health outcomes through its negative impact on women’s mental well-being.

The effects of IPV on the victims are multifaceted including, gastrointestinal disorders, chronic pain, cardiovascular problems, higher rate of depression, low self-esteem, suicidality, vaginal and anal tearing, sexual dysfunction, sexually transmitted disease, pelvic inflammation, low or insufficient gestational weight, antenatal hospitalization, preterm delivery, low birth weight, miscarriages, and neonatal and child mortality [8–12]. Experience of IPV during pregnancy is detrimental to the mother and unborn baby leading to pregnancy-specific behaviors such as not initiating ANC and missing ANC appointments, and adverse birth outcomes such as preterm delivery and low birth weight [13].

ANC often serves as a woman’s first point of entry into a health care system [14]. ANC service is also an opportunity for health professionals not only to encourage women to continue to engage in ANC but also to identify and help women who experience IPV. The routine screening for IPV in the health care setting during ANC visit could open a way for women experiencing IPV to get the psychological and targeted emotional support they need and may help them connect to available resources [15]. It may also help ANC providers ensure that the psychological impact of IPV is not complicating the childbirth and labor process [15].

ANC visits present a unique window of opportunity for women experiencing IPV in which health care providers can foster trusting relationships with the victims that increases the likelihood of IPV detection and mitigation of its negative consequences to both mother and child. However, experience of IPV could limit women’s access to maternal healthcare services reducing women’s opportunity to benefit from such services. Studies from Kenya showed that women who reported IPV also reported insufficient use of ANC services and not having skilled attendance during delivery [16–19]. Victims of IPV likely have reduced uptake of ANC due to social and emotional barriers.

Although there is a renewed commitment from the Mozambican government to bridge the gap in maternal health service use including ANC services, most of the efforts to date are directed toward increasing availability of trained providers and infrastructure, and less so toward understanding the effect of IPV on ANC services utilization. In addition, in 2012, the government of Mozambique developed an integrated mechanism for the care of victims of IPV and gender-based violence by defining more specific roles and responsibilities of various government entities, including the Ministries of Health as related to services to victims of violence [20]. The ultimate goal of this mechanism was to promote integrated services by outlining general guidance for: improving quality of services to IPV and gender-based violence survivors that included a definition of basic procedures of care and development of guidelines and service delivery protocols for all professionals involved in providing services to IPV victims. Although this is a step in a right direction, the effort is faced with major impediments to delivering essential interventions. Some of the impediments include shortage of skilled health professionals, a weak, poorly equipped and fragmented health system, and limited training in culturally appropriate ways of identification and care for victims, as well as lack of forensic capacity. The health sector also faces the challenge of assuring that the integrated services and appropriate forensic medicine services are available, particularly as the law stipulates that without forensic evidence charges against the perpetrators of violence cannot be sustained in the court of justice [7]. This may be due to limited research examining the association between IPV and ANC within the context of Mozambique.

Therefore, addressing this gap is an important next step to inform current and future intervention efforts to increase access to ANC by designing intervention that reduces the effect of IPV both on access to ANC and maternal health outcomes.

The aim of this study was to examine the association between IPV and ANC services utilization in the Sofala province of Mozambique. Using data gathered to assess the impact of membership in savings groups on maternal health service utilization, we evaluated the association between IPV and ANC service utilization among study participants. Specifically, we hypothesize that (1) any form of IPV is associated with lower likelihood of ANC services utilization (at least one ANC and 4+ ANC) and receiving ANC services from skilled personnel; (2) Each form of IPV (e.g. emotional, physical and sexual violence) is associated with lower likelihood of ANC services utilization and receiving ANC services from skilled personnel.

## Method

### Analytic sample

The data for this study was drawn from a larger study conducted as part of an ongoing program progress evaluation in Manga Laforte, Sofala province of Mozambique. The original study sampled women of reproductive age residing in three sub-villages in Manga Laforte with a total population of 64,626. The original study assessed whether membership in Savings Groups (SG)-a community-based microfinance that focused on poor households- was associated with maternal health service utilization and whether the association is mediated by women’s agency. The original study used a two-stage cluster sampling strategy to select SG members and non-members to the study. For the members of SG, we randomly selected 105 female respondents out of a total 485 female SG members in 31 SGs in three sub-villages. The number of SG members sampled per SG was proportional to the total numbers of SG members in each SG. For the comparison group, we randomly selected 30–35 female respondents from each sub-village for a total of 100 and the three sub-villages were treated as clusters. We determined the sample size based on a 5% level of significance (type I error, α = 0.05), power of 80% (type II error, β = 0.20), and a design effect of 2.0. We calculated effect size based on a national average, where 51.7% of females reported that they received ANC services [6].

We adapted a questionnaire from a previously validated Demographic and Health Survey tool [6]. We translated the questionnaire into Portuguese and translated back to English to validate the quality of translation. We trained four enumerators and one supervisor on basic human subject research and the use of the survey instrument. We pilot tested the tool and reviewed for the final use.

### Measures

#### Outcome variables

The outcome variables for this study were ANC visits and ANC from skilled personnel. First, we assessed whether respondents reported receiving ANC services for their most recent pregnancy. For those who reported receiving ANC services, we further assessed the number of ANC visits they received. We used the number of visits as a continuous variable, and we created categories based on WHO guidelines, which recommend at least one and four or more visits [21]. We asked respondents “who they received ANC services from” and those who reported that they “received ANC services from doctor, nurses and other trained health personnel” were coded as 1 and the rest were coded as 0.

#### Primary explanatory variable

Experience of IPV was treated as a primary predictor variable. We assessed experience of IPV through questions related to experience of sexual, emotional and physical violence. In this study, sexual violence is defined as a sexual act committed against someone without that person's freely given consent. We asked the respondent whether, in the last 12-month, respondent’s partner obligated or forced the respondent to have sex with him even though the respondent did not want to. Emotional violence is defined as any act including verbal assault, humiliation, intimidation, infantilization, or any other treatment which may diminish the sense of identity, dignity, and self-worth. Respondent was asked whether her partner, in the last 12 months, said or did something to humiliate the respondent. Lastly, physical violence is defined as the intentional use of physical force or power, threatened or actual harm by her partner. We asked respondent whether, in the last 12 months, her partner had thrown something at/hit/threatened /threatened the respondent with a weapon. These definitions are widely used definitions and concepts of violence within the public health field and the questions are from the previously validated questions used in demographic and health survey [22].

First, participants were presented with questions in three categories: emotional, physical and sexual abuses. Each of these categories were coded 1 for Women who responded ‘yes’ to experience of each form of IPV in the last 12 months and 0 for women who responded “no” to each of the categories, respectively. Finally, we created a new variable, IPV, by combining these categories. IPV was coded as 1 if any of the three categories were coded as 1 and 0 if all the three categories were coded as 0.

### Other covariates

We included other covariates (socio-demographic characteristics) that have been theoretically and empirically proven to be significantly associated with IPV and ANC service utilization in the model. These include: age, educational status, duration of marriage/relationships, parity, membership in a saving group, average income, decision-making autonomy, and rejection of justification of wife beating. We included rejection of wife beating because recent study showed women who strongly reject the justification of wife beating were more likely than those who reject it weakly to report antenatal care, delivery in healthcare facilities, and postnatal care [23]. Education level was coded as 1 for those who did not attend school, 2 for those who attended primary school (grade 1–65), 3 for those who attended middle school (grade 6–8), and 4 for those who attended secondary school and above (grade 9 through college). To facilitate further analysis, respondents who reported attending any level of education were coded as 1 and all other responses were coded as 0.

Respondents’ decision-making autonomy was measured by their involvement in making decisions, alone or with partner, regarding: (a) their personal health care, (b) family planning, (c) large household purchases, (d) loan taking, and (e) savings. These responses were collapsed into a binary variable describing involvement in decision-making in which respondents who reported making decisions alone or jointly with their partners were coded as 1 and those who reported being involved in none of the decision making were coded as 0. The other covariate is rejection of wife beating. Respondents were presented with several scenarios and asked whether they believe that men are justified in beating their wives in any of these scenarios. Respondents who reported “Yes” to at least one of these scenarios were coded as 1 and respondents who reported “No” to all were coded as 0 and we named the variable as “Justifying wife beating”.

### Analysis strategy

First, we computed descriptive statistics and estimated the prevalence of the outcome variables. Second, we assessed differences between women who reported experience of IPV and those who did not report experience of IPV for all outcome variables in the model using Chi-Square tests of association. Next, we tested potential demographic confounders of each outcome in bivariate tests of association. Finally, after assessing the main effects of IPV on ANC service utilization and receiving ANC from skilled personnel, we assessed whether each form of IPV (i.e. sexual, physical and emotional violence) were varied in their association with the outcome of interest. We employed binary logistic regression model controlling for clustering at sub-village level and other confounding variables to assess the association between IPV and ANC use; between IPV and ANC from skilled personnel; between each form of IPV and ANC use; between each for IPV and ANC from skilled personnel. We conducted all analyses using Stata 14.2

### Ethical consideration

The original study which the data for the current study was taken from was conducted as part of an ongoing program progress evaluation and the implementing partner, Oasis Mozambique did not need IRB approval. However, the data collection process followed proper research protocol that included provision of information about the study, obtaining of written informed consent from each study participant before the interview. The original study did not collect personally identifiable information and conducted the interviews in isolated private rooms to ensure both visual and auditory privacy. Although there were two participants with the age of 17 years, both were married or lived with partner at the time of the survey and both consented to participate. Ethical approval for the secondary data analysis was obtained from University of Iowa’s Institutional Review Board.

## Result

We presented descriptive statistics in Table 1. The total number of participants was 205. Most of the respondents were in the age range between 17 and 35 (60.5%) with the mean age of 33.4 years (SD = 11.88, min = 17 and max = 49) and all respondent-including two respondents with age 17 years- were married at the time of the original survey. The proportion of women who reported not having any formal education was 16%. All respondents reported being married (97.6%) or lived with a partner (2.4%), and the mean length of marriage was 6.6 years (SD = 2.40). The mean number of children in the relationship was 3 (SD = 1.43, min = 1 and max = 8). The mean household monthly income was about 3,886 Mozambican meticais (SD = 4336, min = 150 and max = 50000), which was equivalent to 125.00 USD at the time of the survey.

**Table 1:** Descriptive statistics for all study variables(N = 205).

Background Characteristics	ANC service utilization				
	All women N (%)	No ANC from any source n1 (% = n1/N)	At least one ANC n2 (% = n2/N)	Four or more ANC n3 (% = n3/N)	ANC from skilled personnel n4 (% = n4/n2)
Age in years					
17–24	64(31.2)	15(23.4)	49(76.6)	28(43.8)	43(76.7)
25–34	60(29.3)	10(16.7)	50(83.3)	27(45.0)	45(81.8)
35–49	81(39.5)	9(11.1)	72(88.9)	51(63.0)	69(88.5)
Education status					
No formal education	35(17.1)	11(31.4)	24(68.8)	18(50.0)	22(62.8)
Primary school (≤*grade* 5)	74(36.6)	12(16.2)	62(83.8)	37(50.0)	56(75.7)
Middle school (grade 6 to 7)	65(32.2)	9(13.9)	56(86.2)	37(56.9)	53(81.5)
Secondary and above (≥*grade* 8)	31(15.2)	5(16.1)	26(83.9)	16(50.0)	26(83.9)
Duration of marriage/relationship					
≤4	23(11.2)	6(26.1)	17(74.0)	9(39.1)	19(82.6)
>4	182(88.8)	28(15.4)	154 (84.6)	97(53.3)	151(83.0)
Parity					
1	34 (16.6)	10(24.4)	24(70.6)	10(29.4)	26(77.8)
2–3	97(47.3)	16(16.5)	81(83.5)	48(49.5)	79(81.4)
≥4	74(36.1)	8(10.8)	66(89.2)	48(64.9)	64(86.5)
Savings group membership					
Yes	105(51.2)	5(4.8)	100(95.2)	76(72.4)	96(95.1)
No	100(48.8)	29(29)	71(71.0)	30(30.0)	61(61.0)
Household average monthly income (MT)					
Low income(≤2000MT)	71(34.6)	14(20.0)	57(80.0)	30(42.2)	56(78.9)
Middle income (2001–4000 MT)	66(32.2)	13(19.7)	53(80.3)	37(56.1)	52(78.8)
Upper middle income(4001-6000MT)	34(16.6)	4(11.8)	30(88.2)	22(64.7)	30(88.2)
High Income (>6000MT)	34(16.6)	3(9.0)	31(91.0)	17(50.0)	31(91.3)
Women’s decision-making autonomy					
Not involved at all	16(7.8)	7(43.8)	9(56.2)	2(12.5)	9(56.3)
Involved in two	34(16.6)	10(29.4)	24(70.6)	15(44.1)	23(67.6)
Involved in four	56(27.3)	9(16.1)	47(83.9)	32(57.1)	46(82.1)
Involved in all five	99(48.3)	7(7.1)	92(92.9)	57(57.6)	92(91.8)
Disagree to justifying wife beating					
Yes	42(20.5)	7(17.3)	35(83.3)	23(54.8)	28(81.6)
Self-reported sexual IPV					
Yes	41(20.0)	25(61.0)	16(39.0)	13(29.3)	16(39.0)
Self-reported physical IPV					
Yes	75(36.6)	29(39.0)	46(61.0)	28(37.3)	22(29.3)
Self-reported emotional IPV					
Yes	83(40.5)	24(28.9)	59(71.1)	30(36.1)	38(22.9)
At least one IPV					
Yes	97(47.3)	25(25.8)	27(74.2)	43(44.3)	32(33.6)

ANC = Antenatal care. Note: at least one ANC and four or more ANC are not additive.

Among the study participants, 36.6%, 45.5%, and 17.9% reported income-generation activities (e.g. selling fish, vegetable and finished products such as sugar, soap and edible oil), paid employment, and other as their sources of income, respectively. About 48.3% of respondents reported involvement in all of the household decision-making (personal health care, family planning, large household purchases, loan taking, and savings).

Overall, 47.3% of the respondents reported experiencing at least one form of IPV in the last 12 months. The proportion of respondents who reported sexual, physical and emotional violence was 20%, 36.9% and 20%, respectively. A majority of the respondents (72.7%) reported at least one scenario in which they believe men are justified in beating their wives. The result showed that respondents believe that a man is justified in beating his wife or partner if wife or partner cheats (47.8%), goes out without his permission (37.7%), refuses to have sex with him (27.8%), argues with him (23.9%), neglects a child (28%) and burns food (18.1%). The result showed that of the total respondents, 83.4%, and 51.7% reported receiving at least one, four or more ANC, respectively. Of those reported receiving ANC, 95% of them reported receiving ANC from skilled personnel.

Table 2 presents the adjusted logistic regression models performed to assess the association between IPV and ANC services utilization; between IPV and ANC from skilled personnel; between each form of IPV and ANC; and between each form of IPV and ANC from skilled personnel after controlling for other covariates (age, educational status, duration of marriage/relationships, parity, membership in a saving group, average income, decision-making autonomy, and rejection of justification of wife beating).

**Table 2:** Adjusted Odds Ratios (AOR) for the association between different forms of IPV and utilization of ANC services, Manga Laforte in Beira, Mozambique, 2017 (N = 205).

IPV type	At least one ANC AOR (95%CI)	Four or more ANC AOR (95%CI)	ANC from skilled personnel AOR (95%CI)
Emotional	0.13 (0.04–0.43) ***	0.47 (0.19–0.69) **	0.42(0.16–0.90)
Sexual	0.87 (0.27–2.84)	0.40(0.14–0.89) *	1.16(0.35–3.90)
Physical	0.35(0.21–0.79) *	0.34(0.18–0.67) **	0.51(0.19–1.3)
Any form of IPV	0.50(0.01–0.50) **	0.47(0.20–0.86) *	0.32(0.10–0.90) *

IPV intimate partner violence, ANC Antenatal care. The models adjusted for age, educational status, duration of marriage/relationships, parity, membership in savings group, average income, decision-making autonomy, and rejection of justification of wife beating

* p < .05

** p < .01

*** p < .001, response category “No” is a reference category for the type of IPV in the model

Women who reported experiencing emotional abuse by their partners were less likely to have: at least one antenatal visit (adjusted odds ratio [AOR] = 0.13; 95% confidence interval [CI] = 0.04–0.43) and four or more antenatal visits (AOR = 0.47;95%CI = 0.19–0.69) than their counterparts who did not report experience of IPV. The odds of having four or more antenatal visits was less in women who reported experience of sexual abuse compared to those who did not (AOR = 0.40; 95%CI = 0.14–0.89). Women who reported being physically abused by their partner were less likely to have at least one antenatal visit (AOR = 0.35;95%CI = 0.21–0.79) and four or more antenatal visits (AOR = 0.34; 95%CI = 0.18–0.67). Women who experienced at least one of the three types of abuse (emotional, sexual and physical) were less likely to have at least one (AOR = 0.50; 95%CI = 0.01–0.5), four or more ANC (AOR = 0.47; 95% = 0.20–0.86), and to received ANC from skilled (AOR = 0.32;95CI = 0.10–0.90).

## Discussion

To our knowledge, this is the first study reporting the association between intimate partner violence and ANC service utilization in Mozambique. Nevertheless, a small body of recent work has begun to assess the impact of IPV on ANC service use in other settings [24]. Seeking to extend this new evidence base, we conducted a secondary analysis using data from a study conducted to assess membership in SG, women’s agency and maternal health service use in Sofala Mozambique. This study makes two key contributions. First, it presents the association between experience of IPV in Sofala, Mozambique, which may inform future maternal health promotion efforts. Second, this study adds to the emerging literature that suggests that the association between IPV and ANC may follow a bidirectional relationship where an ANC visits leading to the disclosure of IPV experience could trigger relationship conflict leading to more IPV experience, and/or experience of IPV can worsen women’s access to ANC by discouraging women from ANC visit, especially among those who fear or are controlled by their partners, or do not have the freedom of movement necessary to obtain ANC [25].

The overall IPV prevalence among study participants was 47.3%, which is higher than the national average, 40% [6]. This difference could be attributed to the cultural norms, in the study setting and in central Mozambique in general, that may tolerate violence against women [8]. Emotional violence is the most commonly reported form of IPV in this study (40.5%); followed by physical violence (36.6%), and sexual violence (20%), respectively. This trend is consistent with a previous study conducted in Maputo city, the capital of Mozambique, that reported emotional violence as the most common type of IPV followed by physical and sexual violence, respectively [8].

In this study, we found that women’s involvement in decision-making was lower (24%) among those who reported experience of IPV, which may suggest that the low level of ANC among women who had experienced violence could be due to low levels of autonomy. This finding supports previous studies that linked women’s lack of autonomy to both reduced odds of using ANC and giving birth at health facility [26,27]. Our findings showed reporting experience of IPV was associated with receiving ANC from unskilled providers and those who reported experience of IPV were more likely than other women to obtain care from family members, traditional healers and/or traditional birth attendants. However, unlike previous research that reported women who reported experience of IPV may not abandon care altogether but turn to alternative sources [24], our finding shows that, of the women who reported experience of IPV and did not receive ANC from skilled personnel, only 1% reported receiving ANC from other sources. This may, in part, be due to the patriarchal norms where husbands do not permit their wives to attend antenatal appointments for fear that the abuse they inflicted on their wives and or the mark left on their wives due to physical abuse would be discovered and would reflect poorly on themselves [28].

The results of this study highlight multiple potential points of intervention. In areas of pervasive IPV, training traditional birth attendants to provide culturally appropriate ANC that is sensitive to the needs of women who have experienced IPV may help increase uptake of ANC among women who are unable to seek care or are uncomfortable seeking it elsewhere. In addition, initiatives such as empowering women to be able to make decisions about their health and helping women to participate in women self-help group (e.g. savings group) may help the victims of IPV to obtain ANC.

Although this study generated important evidence and bridged the gap in knowledge about association between IPV and ANC utilization in the study area, there are notable limitations that are inherent to the source data. First, data for this study was drawn from a cross-sectional study and we were not able to measure the temporal as well as causal relationship between the variables of interest. However, estimating the association between IPV and ANC is an important first step to guide future maternal health promotion efforts and related research in the study area. To overcome the limitation in the temporal association, the authors encourage data collection at different points in time in future research, which may foster better rapport and help to draw causal inferences. Second, we asked study participants if they had experienced emotional, sexual, and physical violence in the last 12 months and data may have been subjected to recall bias that might have affected reporting of IPV among all respondents. Questions referring to what happened over the course of a year may be subjected to recall bias thereby underreporting the violence that happened in that length of time [29]. Third, some women may not report experiences that they perceive as minor or unimportant or simply do not recall abusive events that happened but were not recent. Selective screening or case-finding process at a health facility or by a clinician might have helped those who had received ANC to recall their exposure differently than those who did not; however, we did not have a way to indicate that.

## Conclusion

The findings of the present study show statistically significant association between IPV and ANC utilization and supports one of the three pathways outlined in World Health Organization conceptual model [5]. Our finding shows that women who experience IPV are unlikely to use ANC services. This has several implications for health care as well as negative implications on child health services. Less access to ANC is associated with poor pregnancy outcomes including low birth weight and preterm birth [30]. Although there is lack of evidence regarding the most effective health care response to address the issues of IPV [31], this finding highlights multiple significant potential points of intervention for health services. In areas where IPV is prevalent, training traditional birth attendants to provide culturally appropriate ANC that is sensitive to the needs of women who have experienced IPV may help increase uptake of ANC among women who are unable to seek care or are uncomfortable seeking it elsewhere. In addition, home-based ANC visits by clinicians may help reach women who, because they fear or are controlled by their partners, do not have the freedom of movement necessary to obtain ANC. Training clinic-based providers to screen for IPV and to refer women who are experiencing IPV to sources of antenatal care that are easily accessible to women with reduced autonomy may also prove helpful.

This finding also points to the need for addressing IPV in the public health setting that includes integrating prevention, screening, and intervention practices into routine public health programs. Such integrated approach can maximize existing resources and improve the quality of services for victims of IPV. Our study shows that experience of IPV disrupts continuum of care to life saving services. Therefore, it is important that the department of public health in the study area focuses on early identification and intervention that provides an opportunity to educate clients about the continuum of violence which typically escalates over time and the health implications. It is also an opportunity to strategize with clients to identify ways to prevent health effects of abuse on utilization of ANC and how to access preventive care in a way that will be safe for the victims. Further work is needed to better understand whether the association between IPV and maternal health care service utilization is a causal association to help guide a comprehensive intervention to reduce both the experience and the impact of IPV.

## Supporting information

S1 Dataset (DTA)

S1 Questionnaire Portuguese. (PDF)

S2 Questionnaire English. (PDF)
