# Supplementary material for: Women’s experience of intimate partner violence and uptake of Antenatal Care in Sofala, Mozambique
Source: PLoS One. 2019 May 24;14(5):e0217407. doi: 10.1371/journal.pone.0217407 (PMC6534299; doi:10.1371/journal.pone.0217407)
Supplement: S1 Questionnaire — (PDF) [file pone.0217407.s002.pdf]

INSTRUÇÃO PARA CONSENTIMENTO INFORMADO

[Imprima uma cópia deste POR INTREVISTADOR] Antes de entrevistar a cada participante, voçê deve ter o consentimento para realizar a entrevista [Por favor entreviste apenas mulheres]. Por favor, leia exactamente o consentimento informado como está escrito. Esta declaração explica a finalidade da pesquisa e a natureza voluntária de participação do entrevistado, e, em seguida, procure a sua colaboração. Depois de ler a declaração, deve marcar o número do questionário no espaço fornecido abaixo do termo de consentimento. Antes de entregá-lo ao seu supervisor no final do dia, assine o fim da página para confirmar que leu a declaração ao entrevistado. Se a mãe não concorda em ser entrevistada, agradeça-lhe pelo seu tempo, e termine a entrevista.
Reporte todas as recusas ao seu supervisor no mesmo dia.

TERMO DE CONSENTIMENTO INFORMADO

Olá. O meu nome é ________________________________, e eu estou trabalhando com __________________. Estamos a realizar uma pesquisa e agradeciamos a sua participação. Eu gostaria de fazer-lhe perguntas sobre o seu rendimento, a sua saúde, a saúde da sua família, a sua rede de apoio social, relações familiares e sua participação no grupo de poupança. Esta informação nos judará a planear melhor os nossos serviços para a comunidade. A pesquisa geralmente leva 45 a 60 minutos para ser concluída. Todas as respostas que voçê der serão confidenciais e não serão compartilhadas com qualquer outra pessoa senão membros da nossa equipe de pesquisa.

A participação nesta pesquisa é voluntária e voçê pode optar por não responder a qualquer pergunta individual ou a todas as perguntas. Nenhum serviço lhe será retido se optar por não participar. No entanto, esperamos que trá participar nesta pesquisa uma vez que os seus pontos de vista são importantes.

No caso de precisar mais informações sobre a pesquisa, pode entrar em contato com a pessoa listada no cartão que já foi entregue à sua família, quando convidados a participar.

Neste momento, voçê quer perguntar-me qualquer coisa acerca da pesquisa? [*Responda a qualquer pergunta que a mãe tiver*.] Concorda que seja entrevistada? [Se a entrevistada concorda, escreva o número do questionário abaixo e prossiga com a entrevista. Se não concorda, termine a entrevista e procure outra respondente.]

{Se concorda] Número do questionário: ______/__________/____________

[De forma educada peça a participante para assinar o consentimento no espaço abaixo]

[Se concorda] **Assinatura da participante**

___

Assinatura do entrevistador: __________________________________

IDENTIFICAÇÃO(1)			
1. Nome do Local (Distrito): ________________________			
2. Número do Questionário: --->			
	Dia (DD)	Mês (MM)	Ano (YYYY)
3. Data da Entrevista:-->			
4. Nome do Entrevistador:			

INFORMAÇÃO DEMOGRÁFICA (2)

NO.	PERGUNTAS
5	Quantos anos tem? _____________ Anos
6	(Incluindo a si própria) Quantas pessoas vivem na sua casa ou família?<br><br>_____________
7	Daqueles ____________(Resposta a Q6) membros da sua família/casa, quantos são agora:<br><br>1. Idade dos 5 abaixo (0-5 anos de idade)? ____________<br><br>2. Idade dos 5 aos 18 anos de idade? _____________________<br><br>3. Nenhum deles está abaixo dos 18 anos<br><br>9. Não sabe /sem resposta
8.	É membro de Grupo de Poupança?<br><br>1. Sim<br><br>2. Não. ……Salte **para a pergunta 10**
9.	Se for "Sim" para a pergunta 8, Há quanto tempo voçê é membro? __________________

PERGUNTAS SELECIONADAS SOBRE A SAÚDE MATERNO INFANTIL (3)

NO.	QUESTÕES
10	Nos últimos 12 meses, quantas vezes (se é que já) visitou uma unidade de saúde para o cuidar de si ou dos seus filhos?<br><br>1. Nunca Visitou<br><br>2. Uma a duas vezes<br><br>3. Duas a três vezes<br><br>4. Quatro a cinco vezes<br><br>5. Mais do que cinco vezes<br><br>9.Não sabe / sem resposta

<table>
<tr><td>11</td><td colspan="4">Agora gostaria de falar sobre o planeamento familiar - existem várias formas ou métodos que um casal pode usar para impedir ou evitar uma gravidez (por exemplo, implante, comprimido, preservativo, etc).

Está actualmente a fazer algo ou a usar qualquer método para impedir ou evitar a gravidez?

1. Sim

2. Não

9. Não sabe / sem resposta</td></tr>
<tr><td>12</td><td colspan="4">Se for “Sim” para a pergunta 11, por quanto tem vindo a usar (MÉTODO ACTUAL) agora sem parar

1. Mais de um mês mas menos de 6 meses

2. Mais de 6 meses mas menos de um ano

3. Por quase um ano

4. Mais de um ano

9. Não sabe / sem resposta</td></tr>
<tr><td>13</td><td colspan="4">Agora gostaria de perguntar sobre todos os nascimentos que já teve durante a sua vida. Já alguma vez deu à luz?

1. Sim

2. Não ------- Salte para a pergunta 15

9. Não sabe / sem resposta</td></tr>
<tr><td rowspan="3">14</td><td rowspan="3">Se for “Sim”para a pergunta 13, qual é a data de nascimento do seu filho mais novo?</td><td>1.

Dia (DD)</td><td>2.

Mês (MM)</td><td>3.

Ano (YYYY)</td></tr>
<tr><td></td><td></td><td></td></tr>
<tr><td colspan="3">9. Não Sei/Sem Resposta</td></tr>
<tr><td>15</td><td colspan="4">Actualmente está grávida?

1. Sim

2. Não

9. Não sabe / sem resposta</td></tr>
<tr><td>16</td><td colspan="4">Já viu alguém nos cuidados pré-natais na sua gravidez actual ou passada? [Salte esta pergunta se a mãe respondeu “Não” as perguntas 13 e 15]

1. Sim

2. Não. ……Salte para a pergunta 19

9 Não sabe / sem resposta</td></tr>
</table>

<table>
<tr><td rowspan="4">17</td><td colspan="2">Se for “Sim” para a pergunta16, a quém viu? [Resposta múltipla é possível]</td></tr>
<tr><td>Pessoal de Saúde</td><td>Outra Pessoa</td></tr>
<tr><td>1. Médico

2. Enfermeira/Parteira

3. Outro Pessoal de Saúde mas que não conheça a sua especialização</td><td>4. Parteira Tradicional

5. Médico Tradicional/Curandeiro

6. Mães líderes</td></tr>
<tr><td colspan="2">9. Não sabe / sem resposta</td></tr>
<tr><td>18</td><td colspan="2">Se for “Sim” para a pergunta16, quantas vezes já recebeu cuidados pré-natais durante esta gravidez?

1. Uma a duas vezes

3. Três vezes

4. Quatro ou mais vezes

9.Não sabe / sem resposta</td></tr>
<tr><td>19</td><td colspan="2">O tratamento intermitente preventivo da malária na gravidez é um curso terapêutico completo da medicina antimalárico administrado a mulheres grávidas em consultas pré-natais de rotina, independentemente se o destinatário está infectado com a malária. Durante sua gravidez actual ou passada, Já lhe foram dados ou comprou qualquer medicação da malária? [Salte esta pergunta se a mãe respondeu “Não” as perguntas 13 e 15]

1. Sim

2. Não

9.Não sabe / sem resposta</td></tr>
<tr><td>20</td><td colspan="2">(Se houver) Durante toda a gravidez, por quantos meses tomou suplementos de ferro? [Mostrar comprimidos] [Se a entrevistada mencionar os dias, converta para um mês dividindo por 30. [Salte esta pergunta se a mãe respondeu “Não” as perguntas 13 e 15]

1. Nunca ou menos de 15 dias

2. _____ Meses

9. Não sabe / sem resposta</td></tr>
<tr><td>21</td><td colspan="2">Antes de dár à luz o seu filho mais novo, quantas vezes (se houver) recebeu uma injecção no braço para evitar que o bebê apanhasse téctano, isto é, convulsões após o nascimento?

[Salte esta pergunta se a mãe respondeu “Não” as perguntas 13 e 15]

1. Não recebeu injecção no braço

2. Recebeu uma vez

3. Recebeu duas vezes ou mais

9. Não sabe / sem resposta</td></tr>
<tr><td>22</td><td colspan="2">Durante qualquer uma das consultas pré-natais para o seu último nascimento foi oferecido um teste para o vírus</td></tr>
</table>

	da SIDA ou de quaisquer doenças sexualmente transmissíveis, como parte de seus cuidados pré-natais? [Faça entender a entrevistada que não quer saber do resultado]<br><br>**[Salte esta pergunta se a mãe respondeu "Não" as perguntas 13 e 15]**<br><br>1. Sim<br><br>2. Não<br><br>9 Não sabe / sem resposta	
23	Onde que deu parto o seu último filho/a? **[Salte esta pergunta se a mãe respondeu "Não" as perguntas 13 e 15]**<br><br>1. Na Unidade Sanitária<br><br>2. Em Casa<br><br>9. Não sabe / sem resposta	
24	Quem que lhe assistiu no parto do seu último filho/a? **[Salte esta pergunta se a mãe respondeu "Não" as perguntas 13 e 15]**	
	**Pessoal de Saúde**	**Outro Pessoal**
	1. Médico<br><br>2. Enfermeira/Parteira<br><br>3. Outro Pessoal de Saúde mas que não conheça a sua especialização	4. Parteira Tradicional<br><br>5. Médico Tradicional/ Curandeiro<br><br>6. Mães Líderes<br><br>7. Membro de família/Mãe/Sogra/etc
25	Quando o seu bebê mais novo nasceu, quanto ele/a pesava? [verifique o cartão se estiver disponível]**[Salte esta pergunta se a mãe respondeu "Não" as perguntas 13 e 15]**<br><br>1. _______________Kg<br><br>2. Não foi pesado no nascimento<br><br>9. Não sabe / sem resposta	
26	Depois que você deu à luz o seu filho mais novo, quanto tempo será que alguém verificou a sua saúde? **[Salte esta pergunta se a mãe respondeu "Não" as perguntas 13 e 15]**<br><br>1. Ninguém além de membros da família ou parentes que verificaram sobre a sua saúde?<br><br>2. Visitou uma unidade sanitária ou um pessoal de saúde verificou a sua saúde dentro de 48 horas após o nascimento?<br><br>3. Visitou uma unidade sanitária ou um pessoal de saúde verificou a sua saúde após 48 horas, sendo menos de uma semana após o nascimento?<br><br>9. Não sabe / sem resposta	
27	Quanto tempo depois de nascer o seu último bebê deram-lhe a dose de vitamina A? **[Salte esta pergunta se a**	

	**mãe respondeu "Não" as perguntas 13 e 15]**<br><br>1. Antes do bebê ter dois meses de idade<br><br>2. Depois do bebê ter dois meses de idade<br><br>3. A mãe nunca recebeu vitamina A<br><br>9. Não sabe / sem resposta
28	A vacina DPT é uma injecção que é dada na coxa ou nas nádegas, por vezes, ao mesmo tempo que as gotas de pólio. Quantas vezes (se houver) a vacina DPT foi administrada ao seu filho mais novo?<br><br>1. A criança nunca foi vacinada<br><br>2. Uma a duas vezes<br><br>3. Três a quatro vezes<br><br>4. Cinco vezes ou mais<br><br>9. Não sabe / sem resposta
29	A injecção do sarampo ou a injecção MMR - isto é, uma picada no braço com a idade dos 9 meses ou mais - para evitar que as crianças apanhem o sarampo.<br><br>Será que o seu filho/filhos que é/são da idade dos 9 meses ou mais velhos receberam a injecção do sarampo?<br><br>1. Sim<br><br>2. Não
30	Agora eu tenho algumas perguntas sobre o futuro. Depois do bebê que está esperando agora (se estver grávida) ou para além do que você já tem agora, gostaria de ter outro filho, ou iria preferir ter mais filhos?<br>1. Sim quero ter um/outro filho<br>2. Não mais/ Prefiro não mais filhos<br>3. Não consigo engravidar<br>4. Indicisa<br>9. Não sabe / sem resposta
31	Se está indecisa ou quer esperar para ter outro filho, quanto tempo gostaria de esperar a partir de já, antes de nascer de uma/outra criança?<br>1. Mais de seis meses, porém menos de dois anos<br>2. Dois anos<br>3. Mais de dois anos<br>9. Não sabe / sem resposta

32	Agora vou perguntar-lhe se o seu filho ou filhos tiveram diarreia nas últimas duas semanas [Somente pergunte a entrevistada se a sua criança/crianças tiveram diarreia nas últimas duas semanas e saltar esta pergunta se ela disser que nenhum dos seus filhos teve diarreia nas últimas duas semanas]<br><br>Quando o seu filho/filhos tiveram diarreia (possibilidade de Resposta múltipla)<br><br>11. Não fiz nada/ Não dei nada<br><br>12. Dei mais comida para comer<br><br>13. Dei mais do que o habitual para beber<br><br>14. Dei mais leite materno do que o habitual<br><br>15. Dei a mesma quantidade de comida para comer<br><br>16. Dei a mesma quantidade de água para beber<br><br>17. Dei a mesma quantidade de leite materno<br><br>18. Dei SOR/ fluidos caseiros recomendados (água, sumo)<br><br>19. Dei pouca quantidade de comida para comer<br><br>20. Dei pouco para beber<br><br>21. Dei pouca quantidade de leite materno<br><br>9.Não sabe / sem resposta	
33	Você/Seus familiares têm instalaçõessanitárias/latrina?<br><br>1. Sim<br><br>2. Não<br><br>9. Não sabe / sem resposta	
34	Qual é a principal fonte de água de beber para a sua família?	
	11. Canalização dentro da habitação/quintal/terreno	16. Público protegido
	12. Torneira pública	17. Fonte/ rio/ riacho
	13. Poço em casa/quintal/terreno	18. Água de chuva
	14. Poço público	19.Outro(Especifique)________________________
	15. Poço protegido em casa/quintal/terreno	99. Não sabe / sem resposta

35 Na semana passada, fez alguma coisa para a água usada por sua família para torná-la mais segura para beber? Se assim for, o que é?(O que mais?) [Permitidas Múltiplas respostas]

1. Não fiz nada/ Não tratei a água

2. Fervi a água

3. Adicionei lixívia / cloro à água

4. Usei um produto commercial de purificação da água

5. Filtrei atrvéz de um pano fino

6. Utilizei um filtro de água (cerâmica, areia, composto)

7. Usei a desinfecção solar (deixado ao sol)

8. Processo de sedimentação usado (dixado até que sedimentos caiam ao fundo)

9. Outro (Especifique por favor :) ____

36 Quando é lava as mãos com sabão /cinza? (Quando mais?) [Permitidas Múltiplas respostas]

1. Apenas algumas vezes por dia ou por semana

2. Antes de preparar de alimentos

3. Antes de alimentar as crianças

4. Após a defecação

5. Depois de atender a criança que defecou

6. Outros (especifique) ______________________________________

9. Não sabe / sem resposta

37 Agora eu gostaria de lhe perguntar sobre o tipo de alimentos que você ou qualquer outra pessoa em sua casa comeu ontem durante o dia e à noite [Uma nota para o entrevistador: Leia a lista de alimentos e ponha um círculo na resposta]

Grupo de Alimentos	*SIM*	*NÃO*	*Não Sabe*
11. Qualquer [INSIRA QUAISQUER ALIMENTOS LOCAIS ricos em amido, Ex. UGALI, NSHIMA], pão, macarrão de arroz, bolacha, ou qualquer alimento feito de milheto/milho-miúdo, sorgo, milho, arroz, trigo, ou [INSERA QUALQUER OUTRO GRÃO DISPONÍVEL LOCALMENTE]?	1.SIM	0. NÃO	9. NS
12. Qualquer batata, inhame, mandioca ou quaisquer outros alimentos feitos a partir de raízes ou tubérculos?	1. SIM	0. NÃO	9. NS
13. Alguns vegetais? *[Insira exemplos locais]*	1. SIM	0. NÃO	9. NS
14. Algumas frutas? *[Insira exemplos locais]*	1. SIM	0. NÃO	9. NS
15. Qualquer carne de vaca, porco, ovelha, cabrito, coelho de caça selvagem, galinha, pato, ou outras aves, fígado, rim, coração ou outras carnes de órgãos?	1. SIM	0. NÃO	9. NS

16. Alguns ovos?	1. SIM	0. NÃO	9. NS
17. Algum peixe fresco/seco ou marisco?	1. SIM	0. NÃO	9. NS
18. Alguns alimentos feitos a partir de feijão, ervilhas, lentilhas, ou amendoím?	1. SIM	0. NÃO	9. NS
19. Algum queijo, yogurte, leite ou outros produtos feitos de leite?	1. SIM	0. NÃO	9. NS
20. Aluguma comida feita de óleo, gordura ou mateiga?	1. SIM	0. NÃO	9. NS
21. Algum mel ou açúcar?	1. SIM	0. NÃO	9. NS
22. Algum outro tipo de alimentos, tais como tempero, café, chá?	1. SIM	0. NÃO	9. NS
Pontuação da Diversidade Alimentar da Familia (Marque todas as respostas SIM)	Pontuação: _____		

38

Gostaria de lhe perguntar sobre a sua experiência e da sua família em relação a fome	*Nunca*	*Raramente*	*Algumas vezes*	*Muitas vezes*
Durante os últimos trinta dias, quantas vezes passou um dia inteiro e noite sem comer?	*0*	*1*	*1*	*2*
Durante os últimos trinta dias, quantas vezes você ou alguém da sua família dormiu à noite a fome?	*0*	*1*	*1*	*2*
Durante os últimos trinta dias, quantas vezes você não teve qualquer tipo comida em sua casa?	*0*	*1*	*1*	*2*
Pontuação Individual da Fome na Família	__________ *Pontos*			

39

Agora vou ler para si várias afirmações. Eu quero que me diga se concorda totalmente, concorda, discorda ou discorda totalmente com cada uma.

*[**Uma NOTA para o Entrevistador**: Para cada afirmação abaixo, Leia e depois pergunte se a pessoa CONCORDA ou DISCORDA com ela. Se concorda, pergunte,* "Concorda ou Concorda totalmente?"*Se discorda, pergunte,* "Discorda ou discorda totalmente [*Ponha círculo somente numa resposta para cada afirmação]*

Opinião	Discorda Totalmente	Discorda	Concorda	Concorda Totalmente
1. Em situações críticas, prefiro pedir conselhos as outras pessoas.	1	2	3	4
2. Sempre que estou em baixo, procuro alguém para me animar novamente.	1	2	3	4
3. Quando estou preocupado, procuro alguém para conversar.	1	2	3	4
4. If I do not know how to handle a situation, I ask others what they would do.	1	2	3	4
5. Sempre que preciso de ajuda, eu peço.	1	2	3	4
Apoio Social-Buscando Pontuação (Adicionar todos números ciculados)	Pontuação Total: _____ pontos			

40 As declarações a seguir descrevem como as pessoas às vezes sentem sobre si mesmas. Para cada pergunta, por favor, indique quantas vezes sentiu-se assim durante a semana passada.[*Ponha círculo somente numa resposta para cada afirmação]*

Ponha círculo na célula apropriada depois de ler a pergunta abaixo	Raramente ou nenhuma das vezes (0 dias por semana)	Algumas ou um pouco do tempo (1-2 por semana	Ocasionalmente ou uma quantidade moderada de tempo (3-4 dias por semana)	Muitas ou todas as vezes (5-7 dias por semana)	Pontuação (Coloque círculos numerados aqui)
a. Durante a semana passada, em quantos dias sentiu-se muito triste ou deprimida?	4	3	2	1	
c. Durante a semana passada, em quantos dias sentiu-se medrosa?	4	3	2	1	
d. Durante a semana passada, em quantos dias sentiu-se culpada [como uma pessoa má]?	4	3	2	1	
e. Durante a semana passada, em quantos dias sentiu-se cansada o tempo todo?	4	3	2	1	
f. Durante a semana passada, em quantos dias sentiu-se inútil?	4	3	2	1	
g. Durante a semana passada, em quantas noites teve dificuldade em adormecer ou manter o sono?	4	3	2	1	
Pontuação da Depressão<br>*(Adicionar todos os números circulados. Ao contrário, o maior é melhor.)*	Pontuação Total: __________ Pontos				

PERGUNTAS SELECCIONADAS SOBRE EDUCAÇÃO(4)

<table>
<tr><th>NO.</th><th colspan="4">PERGUNTA</th></tr>
<tr><td>41</td><td colspan="4">Qual é o grau mais alto ou ano de escolarida de que já tenha concluído até esse nível?

1. Não frequentou a escola

2. Escola Primária (crechea 5ª classe)/ dois a 7 anos

3. Ensino Básico (6-8ª classe) /8 a10 anos</td></tr>
<tr><td rowspan="7">42</td><td colspan="4">Gostaria de perguntar-lhe sobre a cada um dos seus filhos, sua idade e se vão a escola [Se todas as crianças no agregado familiar tem mais de 18 anos salte esta pergunta]</td></tr>
<tr><td>Nome da criança</td><td>Género</td><td>A criança tem idade escolar?</td><td>A criança está matriculada na escola?</td></tr>
<tr><td>A. Nome da 1ª criança:</td><td>1. Homen

2. Mulher</td><td>1. Sim

2. Não</td><td>1. Sim

2. Não</td></tr>
<tr><td>B. Nome da 2ª criança:</td><td>1. Homem

2. Muher</td><td>1. Sim

2. Não</td><td>1. Sim

2. Não</td></tr>
<tr><td>C. Nome da 3ª criança:</td><td>1.Homen

2.Mulher</td><td>1. Sim

2. Não</td><td>1. Sim

2. Não</td></tr>
<tr><td>D. Nome da 4ª criança:</td><td>1. Homen

2. Mulher</td><td>1. Sim

2. Não</td><td>1. Sim

2. Não</td></tr>
<tr><td>E. Nome da 5ª criança:</td><td>1. Homen

2. Mulher</td><td>1. Sim

2. Não</td><td>1. Sim

2.Não</td></tr>
<tr><td>43</td><td colspan="4">Qual é o nível que diria suficiente para que alguém pudesse ser sucedido hoje?

1. Escola Primária

2. Escola Secundária

3. Técnica / Escola de Comércio

4. Colégio / universidade

5. Pós-graduação ou superior

9. Não sabe / sem resposta</td></tr>
<tr><td>44</td><td colspan="4">Muitos factores diferentes podem impedir que as famílias mandem seus filhos à escola. Quando quiser mandar seu filho / filhos para a escola, qual das seguintes é grande problema [Múltiplas respostas são possíveis

1. Falta de alguém para cuidar dos irmãos menores

2. Ter permissão do meu cônjuge

3. Ter dinheiro necessário para material escolar e mensalidades

4. A distância para o Centro de Saúde.</td></tr>
</table>

	5. Nenhuma das afirmações é problema por enquanto.<br><br>9. Não sabe / sem resposta
45	Quantas crianças no seu agregado familiar não podem frequentar a escola regularmente (ou desistiram), devido à falta de dinheiro (taxa de matrícula, livros de escola, precisam de trabalhar, etc)<br>______________________________

PERGUNTAS SOCIOECONÓMICAS, DE COSMOVISÃO E DE RELACIONAMENTOS

NO.	PERGUNTA	
46	No último mês, qual foi o rendimento aproximado em dinheiro do seu agregado familiar?_________________(MT)	
47	Por favor indique as fontes de rendimento familiar?<br>1. Salário de emprego<br>2. Actividade de geração de renda familiar<br>3.Venda de produtos<br>4. Algumas outras fontes. Especifique_______________________________<br>9. Não sabe / sem resposta	
48	No último mês, a sua família fez qualquer poupança? [se for"Sim" pergunte**o tipo de [poupança e montante**"]<br><br>1. Sim…………**Pergunte os tipos de poupança e montantes**<br>2. Não………. **Salte para a pergunta 49**	
	**Tipos de Poupanças**	**Montante em MT**
	A. Grupo de Poupança	
	B. Banco	
	C. Cooperativa, associação profissional ou produtor SG	
	D. Dentro de casa (de baixo da cama, lata, etc.)	
	E. Empréstimo a um amigo ou parente	
49	Agora gostaria de lhe perguntar sobre a estructura da sua casa [Nota: Escolha o código correspondente correcto]	
	**Estructura**	**Código**
	A. Chão	1=Dirt; 2=Cimento; 3=Cerâmica; 4=Outro
	B. Parede	1=Lama; 2=Tijolo não queimado; 3=Bloco; 4=Cimento; 5=Ferro ondulado; 6=Cimento; 7=Textura; 8=Outro
	C. Janelas	1=Sem janelas; 2=Persianas de madeira; 3=Persianas de metal; 4=Janelas de vidro; 5=Outro
	D. Tecto	1=Textura; 2=Vidors/Folhas; 3=Palha; 4=Ferro ondulado; 5=Cimento; 6=Azulejo; 7=Outro
50	Em média, quanto é que a sua familia gasta por semana em comida?___________(MT)	
51	Em média, quantas refeições você ou membros da família têm por dia? [Frequência da refeição principal, não incluindo lanches]: ____________________________(Média de refeições por dia)	
52	No ano passado, quanto a sua família gastouaproximadamente em serviços de saúde?_________ (MT)	
53	No ano passado, quanto a sua família gastou aproximadamente em serviços de educação (adultos e crianças)?_____________(MT)	
54	Quantas vezes costuma conversar com amigos ou membros da família alargada que vivem fora do seu agregado familiar: Pelo menos diariamente, semanalmente, mensalmente, menos de uma vez por mês, ou nunca?	

	1. Pelo menos diariamente<br><br>2. Pelo menos semanalmente<br><br>3. Pelo menos mensalmente<br><br>4. Menos de uma vez por mês/nunca<br><br>9. Não sabe / sem resposta
55	Como descreveria seu relacionamento com os outros e com a sua família?<br><br>[Não leia a lista mas pergunte e marque a resposta próximo das seguintes alternativas]<br><br>1. Sente que é respeitada na sua família?<br><br>2. Sente que é respeitada na sua comunidade?<br><br>2. Sente que não é respeitada pela sua família e a comunidade?<br><br>9. Não sabe / sem resposta
56	Quanto feliz é na sua relação cm seu esposo/parceira***? [Se actualmente estiver casa/num relacionamento de união*] → *Salte para a pergunta 59***<br><br>1. De modo nenhum / Infeliz<br><br>2. Um pouco feliz (um pouco)<br><br>3. Muito feliz<br><br>4. completamente feliz
57	Em quantos dias numa semana o seu marido / parceiro geralmente briga consigo: Quase que nunca, 1-2 dias por semana, ou quase todos os dias?<br><br>1. Nunca / Quase que nunca<br><br>2. Um ou dois dias por semana<br><br>3. Quase todos os dias<br><br>9. Não sabe / sem resposta
58	Nos últimos 12 meses, será que o seu marido / parceiro / namorado já :<br><br>a. Disse ou fez algo que lhe humilhou? ……………………………………1. Sim 2. Não 9. NS/NR<br><br>b. Lançou-lhe alguma coisa?..1. Sim 2. Não 9. NS/NR<br><br>c. Bateu-lhe? ……………..1. Sim 2. Não 9. NS/NR

	d. Ameaçou-lhe?...1. Sim 2. Não 9. NS/NR<br><br>e. Ameaçou-lhe com uma arma?...1. Sim 2. Não 9. NS/NR<br><br>f. Obrigou ou forçou-lhe a ter sexo com ele mesmo você não querendo?...,,,.1. Sim 2. Não 9. NS/NR<br><br>Total de respostas marcadas. SIM: ____
59	Às vezes, o marido / parceiro fica irritado ou zangado com as coisas que sua esposa / parceira faz. Na sua opinião, quais das seguintes situações o marido ou parceiro tem se justificado em espancar ou bater em sua esposa / parceira:<br><br>a. Se ela sai para fazer algo sem lhe informar? ……………...…….…………..1. Sim 2. Não 9. NS/NR<br><br>b. Se ela negligencia os filhos? ……………………..……………………….1. Sim 2. Não 9. NS/NR<br><br>c. Se ela discute com ele?...………………………1. Sim 2. Não 9. NS/NR<br><br>d. Se ele se recusa a dormir/fazer sexo com ele? ……………………………... 1. Sim 2. Não 9. NS/NR<br><br>e. Se ele queima a comida? ………………………………………………..1. Sim 2. Não 9. NS/NR<br><br>f. Se dorme com o ountro homem?........................ ……………………………1. Sim 2. Não 9. NS/NR<br><br>g. Alguma outra razão?... ……………………1. Sim 2. Não 9. NS/NR<br><br>Total de respostas marcadas. SIM: ____
60	*Diga: Agora eu vou ler para si várias declarações. Eu quero que me diga se você concorda totalmente, concorda, discorda ou discorda totalmente com cada uma.*

Para cada declaração abaixo, leia a declaração e, em seguida, pergunte se pessoa CONCORDA com ela ou NÃO. Se ela concordar, pergunte: "Concorda ou concorda totalmente?" Se ela não concordar, pergunte: "Discorda ou Discorda totalmente?" Círculo apenas uma resposta para cada afirmação.

Opinião	Concorda totalmente	Discorda	Concorda	Concorda totalmente
1. Sempre consigo resolver problemas dificeis se eu tentar mais duro.	1	2	3	4
2. Se alguém opôr se a mim, consigo achar meios e caminhos para encontrar o que peciso	1	2	3	4
3. Quando sou confrontado pelos problemas, consigo encontrar várias soluções	1	2	3	4
4. Posso conseguir resolver muitos problems se eu imprimir o esforço necessário.	1	2	3	4
5. Consigo manter-me calmo quando enfrento dificuldades	1	2	3	4
Pontuação Geral da Auto-eficácia	Pontuação Total: _____ Pontos			

61	Recebeu o pagamentono no último ciclo de poupança? Se sim, quais foram as três mais importantes utilizações do pagamento?<br><br>1. Uso primário do empréstimo: ______________________________<br><br>2. Uso secundário do empréstimo: ____________________________<br><br>3. Uso terciário do empréstimo: ______________________________				
62	Levou um empréstimo no último ciclo de poupança? Se sim, quais foram as três mais importantes utilizações do empréstimo?<br><br>1. uso primário do empréstimo: ______________________________<br><br>2. Uso secundário do empréstimo: ____________________________<br><br>3. Uso terciário do empréstimo: ______________________________				
63	Se tiver uma oportunidade de falar com alguém fora seus familiares sobre problemas no trabalho ou em sua família, quém seria ou seriam essas pessoas? [Múltiplas respostas são possíveis]<br>1. Membros do grupo de poupança<br>2. Grupo de membros da família<br>3. Voluntária da Comunidade ou pessoas informadas/Grupos de cuidados voluntários<br>4. Outros amigos<br>5. Membros da equipe Oasis na comunidade<br>9. Outros (Especifique)________________________________				
64	Principalmente, que membro da família faz as seguintes decisões da casa?				
	**Decisões**	**Código**			
	A. Consumo da casa	1=Mulher Chefe de família; 2=Homem Chefe de família; 3=Juntos 5=Outro; 9=Não sei			
	B. Poupanças	1= Mulher Chefe de família ; 2= Homem Chefe de família ; 3= Juntos 5= Outro; 9=Não sei			
	C. Fazer empréstimo	1= Mulher Chefe de família ; 2= Homem Chefe de família ; 3= Juntos 5= Outro; 9=Não sei			
	D. Educação das crianças	1= Mulher Chefe de família ; 2= Homem Chefe de família ; 3= Juntos 5= Outro; 9=Não sei			
	E. Casamento de crianças	1= Mulher Chefe de família ; 2= Homem Chefe de família ; 3= Juntos 5= Outro; 9=Não sei			
	F. Despesas de saúde	1= Mulher Chefe de família ; 2= Homem Chefe de família ; 3= Juntos 5= Outro; 9=Não sei			
	G. Planeamento familiar	1= Mulher Chefe de família ; 2= Homem Chefe de família ; 3= Juntos 5= Outro; 9=Não sei			
65	Qual dos seguintes ativos você possui? [Resposta múltipla é possível]				
	11. Carro<br>12. Motorizada<br>13. Biscicleta<br>14. Carrinha	15. Rádio<br>16. Televisão<br>17.Ventilador/Ar-condicionado<br>18. Antena parabólica	19. Panela de metal<br>20. Congelador<br>21. Micro-ondas<br>22. Máquina de costura	23. Vaca<br>24. Ovelha<br>25. Galinha/Pato<br>26. Cabritos	27. Trator<br>28. Enxada<br>29. Charua<br>30. Bomba Irrigação<br>99. Não sabe / sem resposta

Diga "Obrigado pelo seu tempo"

___________________________FIM____
