# Supplementary material for: Women’s experience of intimate partner violence and uptake of Antenatal Care in Sofala, Mozambique
Source: PLoS One. 2019 May 24;14(5):e0217407. doi: 10.1371/journal.pone.0217407 (PMC6534299; doi:10.1371/journal.pone.0217407)
Supplement: S2 Questionnaire — (PDF) [file pone.0217407.s003.pdf]

INSTRUCTION FOR INFORMED CONSENT

[Print one copy of this PER INTERVIEWER.] Before interviewing each participant, you must get consent to conduct the interview [Please interview only women]. Please read the informed consent exactly as it is written. This statement explains the purpose of the survey and the voluntary nature of the respondent's participation, and then seeks her cooperation. After reading the statement, you should mark the questionnaire number in the space provided below the consent statement. Before turning it into your supervisor at the end of the day, sign the bottom of this page to affirm that you have read the statement to the respondent. If the mother does not agree to be interviewed, thank her for her time, and end the interview. Report all refusals to your supervisor on the same day.

INFORMED CONSENT STATEMENT

Hello. My name is ________________________________, and I am working with __________________. We are conducting a survey and would appreciate your participation. I would like to ask you questions about your income, your health, your family's health, your social support network, family relationships and your participation in the savings group. This information will help us to plan our services to the community better. The survey usually takes 45 to 60 minutes to complete. All of the answers you give will be confidential and will not be shared with anyone other than members of our survey team.

Participation in this survey is voluntary and you can choose not to answer any individual question or all of the questions. No services will be withheld if you chose not to participate. However, we hope that you will participate in this survey since your views are important.

In case you need more information about the survey, you may contact the person listed on the card that has already been given to your household when invited to participate.
At this time, do you want to ask me anything about the survey? *[Answer any questions the mother has.]* Do you agree to be interviewed? [If respondent agrees, write the questionnaire number below and proceed with the interview. If she does not, end the interview and seek another respondent.]

{If agree] Questionnaire Number: ______/__________/____________

[Politely request the participant to sign the consent on the space provided below]

[If agree] **Signature of participants**

Signature of interviewer: ___________________________________

IDENTIFICATION (1)

1. Place Name (District): ____________________________________Group Name:

2. Questionnaire Number:

3. Date of Interview:

Day (DD)	Month (MM)	Year (YYYY)

4. Interviewer's Name:

DEMOGRAPHIC INFORMATION (2)

NO.	QUESTIONS
5	How old are you? ______________ Years
5.1	Marital status<br><br>1. Married<br>2. Single<br>3. Separated/widowed/divorced<br><br>9. Do not know/No Response
5.2	If 1 for Q5.1, For how long you are married?____________
6	(Including yourself) how many people live in your household or family?<br>______________
7	Of those _____________(Response to Q6) members of your family/household, how many are now:<br>1. Age 5 and younger (0-5 yeas old)? _____________<br>2. Age 6 to 18 years old? ______________________<br>3. None of them are below 18 years of age<br>9. Do not know/No Response
8.	Are you a member of Savings group?<br>1. Yes<br>2. No. ……Skip **to question 10**
9.	If "Yes" for question 8, how long have you been a member? ___________________

SELECTED MATERNAL AND CHILD HELATH QUESTIONS (3)

NO.	QUESTION
10	In the last 12 months, how many times (if any) have you visited a health facility for care for yourself or your children?<br>1. Never visited<br>2. One to two time<br>3. Two three time<br>4. Four to five times<br>5. More than five times<br>9. Do not know/No Response
11	Now I would like to talk about family planning – there are various ways or methods that a couple can use to delay or avoid a pregnancy (for example, implant, pill, condom etc).<br>Are you currently doing something or using any method to delay or avoid getting pregnant?<br>1. Yes<br>2. No

<table>
<tr><td></td><td colspan="4">9. Do not know/No Response</td></tr>
<tr><td>12</td><td colspan="4">If "Yes" to Question 11, For how long have you been using (CURRENT METHOD) now without stopping?
1. More than an month but less than 6 months
2. More than 6 months but less than a year
3. For about one year
4. More than a year
9. Do not know/No Response</td></tr>
<tr><td>13</td><td colspan="4">Now I would like to ask about all the births you have had during your life. Have you ever given birth?
1. Yes
2. No ------- Skip to question 15
9. Do not know/No Response</td></tr>
<tr><td rowspan="3">14</td><td rowspan="2">If "Yes" for Question 11, what is your youngest child's date of birth?</td><td>1.
Day (DD)</td><td>2.
Month (MM)</td><td>3.
Year (YYYY)</td></tr>
<tr><td></td><td></td><td></td></tr>
<tr><td></td><td colspan="3">9. I do not know/No response</td></tr>
<tr><td>15</td><td colspan="4">Are you currently pregnant?
1. Yes
2. No
9. Do not know/No Response</td></tr>
<tr><td>16</td><td colspan="4">Did you see anyone for antenatal care for your current or last pregnancy? [Skip this question if mother responded "No" to both questions 13 and 15]
1. Yes
2. No. ……Skip to question 19
9. Do not know/No Response</td></tr>
<tr><td rowspan="4">17</td><td colspan="4">If "Yes" to Question 16, whom did you see? [Multiple response is possible]</td></tr>
<tr><td colspan="2">Health Personnel</td><td colspan="2">Other Person</td></tr>
<tr><td colspan="2">1. Doctor
2. Nurse/Midwife
3. Other health personnel but do not know his/her specialization</td><td colspan="2">4. Traditional birth attendant
5. Traditional healer/which doctor
6. Mother leaders/CHWs</td></tr>
<tr><td colspan="4">9. Do not know/No Response</td></tr>
<tr><td>18</td><td colspan="4">If "Yes" to question 16, how many times did you receive antenatal care during this pregnancy?
1. One time to two times
3. Three times
4. Four or more times
9. Do not know/No Response</td></tr>
<tr><td>19</td><td colspan="4">Intermittent preventive treatment of malaria in pregnancy is a full therapeutic course of antimalarial medicine given to pregnant women at routine antenatal care visits, regardless of whether the recipient is infected with malaria. During your current or last pregnancy, were you given or did you buy any malaria medication? [Skip this question if mother responded "No" to both questions 13 and 15]
1. Yes
2. No
9. Do not know/No Response</td></tr>
<tr><td>20</td><td colspan="4">(If any) During the whole pregnancy, for how many months did you take Iron supplements? [Show tablets] [If respondent mentions days, convert it to a month by dividing by 30. [Skip this question if mother responded "No" to both questions 13 and 15]
1. Never or less than 15 days</td></tr>
</table>

<table>
<tr><td></td><td colspan="2">2. _____ Months
9. Don’t Know / No Response</td></tr>
<tr><td>21</td><td colspan="2">Before you gave birth to your youngest child how many times (if any) did you receive an injection in the arm to prevent the baby from getting tetanus, that is, convulsions after birth? [Skip this question if mother responded “No” to both questions 13 and 15]
0. Did not receive injection in the arm
1. Received one time
2. Received two times or more
9. Don’t Know / No Response</td></tr>
<tr><td>22</td><td colspan="2">During any of the antenatal visits for your last birth were you offered a test for the AIDS virus or any sexually transmitted diseases as part of your antenatal care? [Let the respondent know that you do not want to know the result] [Skip this question if mother responded “No” to both questions 13 and 15]
1. Yes
2. No
9. Don’t Know / No Response</td></tr>
<tr><td>23</td><td colspan="2">Where did you give birth to your youngest child? [Skip this question if mother responded “No” to both questions 13 and 15]
1. At health facility
2. At home
9. Don’t Know / No Response</td></tr>
<tr><td rowspan="3">24</td><td colspan="2">Who assisted with the delivery of your youngest child? [Skip this question if mother responded “No” to both questions 13 and 15]</td></tr>
<tr><td>Health Personnel</td><td>Other Person</td></tr>
<tr><td>1. Doctor
2. Nurse/Midwife
3. Other health personnel but do not know his/her specialization</td><td>4. Traditional birth attendant
5. Traditional healer/which doctor
6. Mother leaders/CHWs
7. Family member/mother/mother in-law etc.</td></tr>
<tr><td>25</td><td colspan="2">When you youngest child was born, how much did he/she weight? [Check the card if available] [Skip this question if mother responded “No” to both questions 13 and 15]
1. _______________Kg
2. Was not weighed at birth
9. Don’t Know / No Response</td></tr>
<tr><td>26</td><td colspan="2">After you gave birth to your youngest child, how soon did anyone check on your health? [Skip this question if mother responded “No” to both questions 13 and 15]
1. No one other than family members or relative checked on your health
2. You visited a health facility or health personnel checked on your health within 48 hours after birth
3. You visited a health facility or health personnel checked on your health after 48 hours but less than a week after birth
9. Don’t Know / No Response</td></tr>
<tr><td>27</td><td colspan="2">How soon after you gave birth to your youngest child were you given the dose of vitamin A? [Skip this question if mother responded “No” to both questions 13 and 15]
1. Before the child was two months old
2. After the was two months old
3. Mother never received vitamin A
9. Don’t know/no response</td></tr>
<tr><td>28</td><td colspan="2">A DPT vaccination is an injection that is given in the thigh or buttocks, sometimes at the same time as polio drops. How many times (if ay) was the DPT vaccination given to your youngest child?
1. The child never given vaccinated
2. One to two time
3. Three to four times
4. Five times or more
9. Don’t know/no response</td></tr>
</table>

29	A measles injection or an MMR injection - that is, a shot in the arm at the age of 9 months or older - to prevent children from getting measles.<br>Did you child/children who is/are age of 9 months or older receive measles injection?<br>1. Yes<br>2. No
30	Now I have some questions about the future. After the child you are expecting now (if pregnant) or in addition to what you now have, would you like to have another child, or would you prefer to have any more children?<br>1. Yes I want to have a/another child<br>2. No more/prefer no children<br>3. I can't get pregnant<br>4. Undecided<br>9. Don't know/No response
31	If you are undecided or want to wait to have another child, how long would you like to wait from now before the birth of a/another child?<br>1. More than 6 months but less than two year<br>2. Two years<br>3. More than two years<br>. Don't know/No response
32	Now I will ask you if your child or children had diarrhea in the last two weeks [Ask the respondent only if her child/children had diarrhea in the last two weeks and skip this question if the she said none of her children had diarrhea in the last two week]<br>When you child/children had diarrhea (multiple response is possible):<br>11. Did not do anything/did not offer anything<br>12. Offered more food to eat<br>13. Offered more than usual to drink<br>14. Offered more breast milk than usual<br>15. Offered same amount of food to eat<br>16. Offered same amount of drink<br>17. Offered same amount of breast milk<br>18. Offered ORS/recommended home fluids (water, juice)<br>19. Offered less amount of food to eat<br>20. Offered less amount to drink<br>21. Offered less amount of breast milk<br>99. Do not know/No response
33	Do you/your family members have toilet facility/latrine?<br>1. Yes<br>2. No<br>9. I do not know/No response

34	What is the main source of drinking water for members of your household	
	11. Piped into dwelling / yard / plot	16. Protected public
	12. Public tap	17. Spring / river / stream
	13. Open well in dwelling / yard / plot	18. Rainwater
	14. Open public well	19. Other (specify)____________________________
	15. Protected well in dwelling / yard / plot	99. Don't know / no response
35	In the past week, did you do anything to the water used by your family to make it safer to drink? If so, what? (What else?) [Multiple answers allowed]<br>1. Did nothing / did not treat the water	

	2. Boiled the water<br>3. Added bleach / chlorine to the water<br>4. Used a commercial water purification product<br>5. Sieved it through a fine cloth<br>6. Used a water filter (ceramic, sand, composite)<br>7. Used solar disinfection (left it in the sun)<br>8. Used sedimentation (left it so sediment falls to the bottom)<br>9. Other (please specify:) ____
36	When do you wash your hands with soap/ash? (When else?) [Multiple answers allowed]<br>1. Only few times a day or a week<br>2. Before food preparation<br>3. Before feeding children<br>4. After defecation<br>5. After attending to a child who has defecated<br>6. Other (specify)________________________________<br>9. Do not know/no response
37	Now I would like to ask you about the types of foods that you or anyone else in your household ate yesterday during the day and at night [note for interviewer: read the list of foods and circle the response]

Food Group	*YES*	*NO*	*Don't Kn*
11. Any [INSERT ANY LOCAL starchy FOODS, E.G. UGALI, NSHIMA], bread, rice noodles, biscuits, or any other foods made from millet, sorghum, maize, rice, wheat, or [INSERT ANY OTHER LOCALLY AVAILABLE GRAIN]?	1. YES	0. NO	9. DK
12. Any potatoes, yams, manioc, cassava or any other foods made from roots or tubers?	1. YES	0. NO	9. DK
13. Any vegetables? *[Insert local examples]*	1. YES	0. NO	9. DK
14. Any fruits? *[Insert local examples]*	1. YES	0. NO	9. DK
15. Any beef, pork, lamb, goat, rabbit wild game, chicken, duck, or other birds, liver, kidney, heart, or other organ meats?	1. YES	0. NO	9. DK
16. Any eggs?	1. YES	0. NO	9. DK
17. Any fresh or dried fish or shellfish?	1. YES	0. NO	9. DK
18. Any foods made from beans, peas, lentils, or nuts?	1. YES	0. NO	9. DK
19. Any cheese, yogurt, milk or other milk products?	1. YES	0. NO	9. DK
20. Any foods made with oil, fat, or butter?	1. YES	0. NO	9. DK
21. Any sugar or honey?	1. YES	0. NO	9. DK
22. Any other foods, such as condiments, coffee, tea?	1. YES	0. NO	9. DK
Household Dietary Diversity Score (Count all Yes responses)	Score: ______		

38 I'd like to ask you about you and your family's experience of hunger

Question	*Never*	*Rarely*	*Some-times*	*Often*
During the past thirty days, how often did you go a whole day and night without eating?	*0*	*1*	*1*	*2*
During the past thirty days, how often did you or someone in your household go to sleep at night hungry?	*0*	*1*	*1*	*2*
During the past thirty days, how often did you not have food to eat of any kind in your household?	*0*	*1*	*1*	*2*
Individual Household Hunger Score:	___________ *Points*			

39 *Now I am going to read to you several statements. I want you to tell me if you strongly agree, agree, disagree, or strongly disagree with each one.*

*[**Note for interviewer**: For each statement below, read the statement and then ask the person if s/he AGREES or DISAGREES with it. If they Agree, ask,* "Do you Agree or Strongly Agree?" *If they Disagree, ask,* "Do you Disagree or Strongly Disagree?" *Circle only one response for each statement.*

Belief	Strongly Disagree	Disagree	Agree	Strongly Agree
1. In critical situations, I prefer to ask others for their advice.	1	2	3	4
2. Whenever I am down, I look for someone to cheer me up again.	1	2	3	4
3. When I am worried, I reach out to someone to talk to.	1	2	3	4
4. If I do not know how to handle a situation, I ask others what they would do.	1	2	3	4
5. Whenever I need help, I ask for it.	1	2	3	4
Social Support-Seeking Score (Add all circled numbers.)	Total Score: _____ points			

40 The following statements describe how people sometimes feel about themselves. For each question, please indicate how often you have felt this way during the past week. [*Circle only one response for each statement]*

Circle the appropriate cell after reading the question below	Rarely or none of the time (0 days a week)	Some or a little of the time (1-2 days a week)	Occasionally or a moderate amount of time (3-4 days a week)	Most or all of the time (5-7 days a week)	Score (Put numbered circled here
a. Over the past week, on how many days did you feel very sad?= E_8	4	3	2	1	
b. Over the past week, on how many days did you feel depressed?= E_4	4	3	2	1	
c. Over the past week, on how many days did you feel fearful or scared? = E_5	4	3	2	1	
d. Over the past week, on how many days did you feel guilty [like a bad person]? =E_3	4	3	2	1	
e. Over the past week, on how many days did you feel tired all the time?= E_6	4	3	2	1	
f. Over the past week, on how many days did you feel worthless that made you unhappy? =E_9	4	3	2	1	
g. Over the past week, on how many nights did you have trouble falling asleep or staying asleep? = E_7	4	3	2	1	
Depression Score (Add all circled numbers. Inverse, higher is better.)	Total Score: __________ points				

SELECTED EDUCATION QUESTIONS (4)

NO.	QUESTION			
41	What is the highest grade or year of school you have completed at that level?<br>1. Did not attend school<br>2. Primary school (infantile to 5th grade)/ two to 7 years<br>3. Middle school (6-8th grade) /8 to 10 years<br>4. Secondary (9-12th)/11 to 14 years<br>5. Tertiary (vocational, colleges, university)/more than 14 years<br>9. Do not know/No Response			
42	I'd like to ask you about each of your children, their age and whether they are going to school ***[If all children in the household are older than 18 years skip this question]***			
	Name of Child	Gender	Is child school aged?	Is child registered in school?
	A. Child #1's name:	1. Male<br>2. Female	1. Yes<br>2. No	1.Yes<br>2. No
	B. Child #2's name:	1. Male<br>2. Female	1. Yes<br>2. No	1.Yes<br>2. No
	C. Child #3's name:	1. Male<br>2. Female	1. Yes<br>2. No	1.Yes<br>2. No
	D. Child #4's name:	1. Male<br>2. Female	1. Yes<br>2. No	1.Yes<br>2. No
	E. CHILD #5's name:	1. Male<br>2. Female	1. Yes<br>2. No	1.Yes<br>2. No
43	What level of education would you say is sufficient in order for one to be successful today?<br>1. Primary school<br>2. Secondary School<br>3. Technical / trade school<br>4. College / university<br>5. Post-graduate or higher<br>9. Don't Know / no response.			
44	Many different factors can prevent families from sending their children to school. When you want to send your child/children to school, which of the following is big problem? [Multiple response is possible]<br>1. Lack of someone to take care of younger siblings<br>2. Getting permission from my spouse<br>3. Getting money needed for school material and fees<br>4. The distance to the health facility<br>5. None of the above is a big problem now<br>9. Don't Know / no response.			
45	How many children in your household cannot attend school regularly (or have dropped out) due to a lack of money (tuition, schoolbooks, need to work, etc.)<br>________________________________			

SELECTED SOCIOECONOMIC, WORLDVIEW, AND RELATIONSHIPS QUESTIONS

NO.	QUESTION
46	In the last month, what was the approximate CASH INCOME of your household?<br>__________________(MT)
47	Please indicate the sources of household income?<br>1. Salary from employment<br>2. Household income-generating-activity<br>3. Sales of produce<br>4. Some other sources. Specify_______________________________

	9. I do not know/No response	
48	In the last month, did your household make any SAVINGS? [If "yes" ask **type of saving & amount**"]<br><br>1. Yes………..Ask the **type of savings and amounts**<br>2. No………. **Go to question 49**	
	**Type of savings**	**Amount in MT**
	Savings group	
	Bank	
	Cooperative, professional association or producer SG	
	In the house (under the bed, tin, etc.)	
	Loan to a friend or relative	
49	Now I'd like to ask you about the structure of your house [Note: Choose the correct corresponding code]	
	**Structure**	**Code**
	Floor	1=Dirt; 2=Cement; 3=Ceramic; 4=Other
	Wall	1=Mud; 2=Un-burnt brick; 3=Brick; 4=Cement; 5=Corrugated Iron; 6=Cement; 7=Fabric; 8=Other
	Windows	1=No windows; 2=Wooden shutters; 3=Metal shutters; 4=Glass windows; 5=Other
	Roof	1=Fabric; 2=Grass/leaves; 3=Straw; 4=Corrugated Iron; 5=Cement; 6=Tile; 7=Other
50	On average, how much does your household expend per week on FOOD?<br>_________________(MT)	
51	On average, how many meals do you or family members eat per day? [Frequency for mail meal time and do not including snacks]: __________________________(average number of meal per day)	
52	In the last year, approximately how much did your household expend on health services?<br>________________ (MT)	
53	In the last year, approximately how much did your household expend on education (adults and children)?<br>____________________(MT)	
54	How often do you usually talk with friends or extended family members who live <u>outside</u> of your household: At least daily, at least weekly, at least monthly, less than once a month, or never?<br>1. At least daily<br>2. At least weekly<br>3. At least monthly<br>4. Less than once a month/never<br>9. Don't know / No response	
55	How would you describe your relationships with others and with your family? [Do not read the list ask the question and mark the response closer to the following alternatives]<br>1. You feel you are respected in your household<br>2. You feel your are respected in your community<br>2. You feel you are not respected by both your family and your community<br>9. I do not know/No response	

56	How happy are you with your relationship with your husband/partner***? [If not currently in marriage/union relationship →Skip to question 59***<br>1. Not at all / unhappy<br>2. Somewhat happy (a little bit)<br>3. Mostly happy<br>4. Completely happy
57	On how many days out of a week does your husband/partner usually quarrel with you: Hardly ever, 1-2 days a week, or almost every day?<br>1. Never / Hardly Ever<br>2. One or two days a week<br>3. Almost every day<br>9. Don't know/ No response
58	In the last 12 months, has your husband/partner/boyfriend:<br>a. Said or done something to humiliate you? …………………………………1. Yes 2. No 9. DK/NR<br>b. Thrown something at you?.. 1. Yes 2. No 9. DK/NR<br>c. Hit you? ……………..1. Yes 2. No 9. DK/NR<br>d. Threatened you?..1. Yes 2. No 9. DK/NR<br>e. Threatened you with a weapon?..1. Yes 2. No 9. DK/NR<br>f. Obligated or forced you to have sex with him even though you didn't want to?..1. Yes 2. No 9. DK/NR<br>Total answers marked YES: ____
59	Sometimes a husband/partner is annoyed or angered by things which his wife / partner does. In your opinion, in which of the following situations is a husband or partner <u>justified</u> in hitting or beating his wife/partner:<br>a. If she goes out to do something without telling him? …………....………1. Yes 2. No 9. DK/NR<br>b. If she neglects the children? ……………………..………………………1. Yes 2. No 9. DK/NR<br>c. If she argues with him?...………………………1. Yes 2. No 9. DK/NR<br>d. If she refuses to sleep with / have sex with him? ………………………….. 1. Yes 2. No 9. DK/NR<br>e. If she burns the food? ……………………………………………….……1. Yes 2. No 9. DK/NR<br>f. If she sleeps with another man?........................ ……………………………1. Yes 2. No 9. DK/NR<br>g. Any other reason?.. …………………… 1. Yes 2. No 9. DK/NR<br>Total responses marked YES: ____
60	*Say: Now I am going to read to you several statements. I want you to tell me if you strongly agree, agree, disagree, or strongly disagree with each one.*

For each statement below, read the statement and then ask the person if s/he AGREES or DISAGREE with it. If they Agree, ask, "Do you Agree or Strongly Agree?" *If they Disagree, ask,* "Do you Disag or Strongly Disagree?" *Circle only one response for each statement.*

Belief	Strongly Disagree	Disagree	Agree	Strongly Agre
1. I can always manage to solve difficult problems if I try hard enough.	1	2	3	4
2. If someone opposes me, I can find the means	1	2	3	4

	and ways to get what I want.				
	3. When I am confronted with a problem, I can find several solutions.	1	2	3	4
	4. I can solve most problems if I invest the necessary effort.	1	2	3	4
	5. I can remain calm when facing difficulties.	1	2	3	4
	Generalized Self-Efficacy Score (Add all circled numbers.)	Total Score: _____ points			

61	Did you receive pay out in the last saving cycle? If Yes, what was the three most important use of pay out?<br>1. Primary use of loan: ________________________________<br>2. Secondary use of loan: ______________________________<br>3. Tertiary use of loan: ________________________________
62	Did you take a loan in the last saving cycle? If Yes, what was the three most important use of the loan?<br>1. Primary use of loan: ________________________________<br>2. Secondary use of loan: ______________________________<br>3. Tertiary use of loan: ________________________________
63	If you get chances to talk to someone outside your family members about problems at work or in your family, who that person or persons would be? [Multiple responses are possible]<br><br>1. Savings group members<br>2. Family groups member<br>3. Community Volunteers or Cascade/Care Groups volunteers<br>4. Other friends<br>5. Oasis staff members in the community<br>9. Others (specify)__________________________________
64	Primarily, which household member makes the following household decisions?

	**Decisions**	**Code**
64	Household Consumption	1=Female head of household; 2=Male head of household; 3=together 5=Other; 9=Do not know
	Savings	1=Female head of household; 2=Male head of household; 3=together 5=Other; 9=Do not know
	Loan-taking	1=Female head of household; 2=Male head of household; 3=together 5=Other; 9=Do not know
	Children Education	1=Female head of household; 2=Male head of household; 3=together 5=Other; 9=Do not know
	Children marriage	1=Female head of household; 2=Male head of household; 3=together 5=Other; 9=Do not know
	Health expense	1=Female head of household; 2=Male head of household; 3=together 5=Other; 9=Do not know
	Family Planning	1=Female head of household; 2=Male head of household; 3=together 5=Other; 9=Do not know

65	Which of the following assets you own? [Multiple response is possible				
	11. Car<br>12. Motorcycle<br>13. Bicycle<br>14. Cart	15. Radio<br>16. Television<br>17. Fan/air conditioner<br>18. Satellite Dish	19. Metal cooking pot<br>20. Refrigerator<br>21. Microwave<br>22. Sewing machine	23. Cow<br>24. Sheep<br>25. Chicken/Duck<br>26. Goats	27. Tractor<br>28. Hoe<br>29. Plough<br>30. Irrigation pump<br>99. Do not know/No response

Say "Thank you for your time"

____________________________END____
